# Retroviral Vectors: Post Entry Events and Genomic Alterations

**DOI:** 10.3390/v3050429

**Published:** 2011-04-29

**Authors:** Ali Nowrouzi, Hanno Glimm, Christof von Kalle, Manfred Schmidt

**Affiliations:** 1 Department of Translational Oncology, German Cancer Research Center, Im Neuenheimer Feld 581, 69120 Heidelberg, Germany; E-Mail: ali.nowrouzi@nct-heidelberg.de (A.N.); 2 National Center for Tumor Diseases, Im Neuenheimer Feld 581, 69120 Heidelberg, Germany

**Keywords:** retroviral vectors, retroviral integration, gene therapy, insertional mutagenesis, vector genotoxicity, Leukemia, common insertion sites

## Abstract

The curative potential of retroviral vectors for somatic gene therapy has been demonstrated impressively in several clinical trials leading to sustained long-term correction of the underlying genetic defect. Preclinical studies and clinical monitoring of gene modified hematopoietic stem and progenitor cells in patients have shown that biologically relevant vector induced side effects, ranging from *in vitro* immortalization to clonal dominance and oncogenesis *in vivo*, accompany therapeutic efficiency of integrating retroviral gene transfer systems. Most importantly, it has been demonstrated that the genotoxic potential is not identical among all retroviral vector systems designed for clinical application. Large scale viral integration site determination has uncovered significant differences in the target site selection of retrovirus subfamilies influencing the propensity for inducing genetic alterations in the host genome. In this review we will summarize recent insights gained on the mechanisms of insertional mutagenesis based on intrinsic target site selection of different retrovirus families. We will also discuss examples of side effects occurring in ongoing human gene therapy trials and future prospectives in the field.

## 1. Introduction

The defining feature of retroviral replication is the integration of the reverse transcribed viral DNA into the genome of the host cell [1,2]. Efficient integration of the viral DNA into the host genome is a hallmark of the retroviral life cycle making replication-incompetent retroviral vectors attractive gene transfer vehicles for stable ectopic expression of transgenes in target cells. The understanding of basic principles of the retroviral replication cycle has led to the development of replication incompetent retroviral vectors capable of a single integration event upon infection of target cells in the absence of superinfection [3–6]. Since their first description in the beginning of the 1980s, a variety of vector systems from the two different retroviral subfamilies—*Orthoretrovirinae* and *Spumaretrovirinae*—have been developed, which are broadly applied in basic and clinical research as ectopic gene delivery vehicles [7,8]. The viral integration reaction catalyzed by the viral integrase protein has been extensively analyzed revealing complex interactions with cellular host proteins regulating nuclear import, chromatin tethering and integration into the host genome [9,10].

Being applied in >357 initiated clinical phase I/II gene therapy trials, retroviral based vectors represent the second most commonly used gene delivery vehicles after adenoviral vectors [11]. Stable integration into the host genome and subsequent long-term ectopic expression of therapeutic transgenes underlines the potential of retroviral gene transfer systems for correction of inherited diseases [12].

Gene therapy for inherited diseases has demonstrated that modification and retransplantation of gene corrected (stem) cells cures severe disorders. The success of these gene therapy trials has been accomplished by replacing genetically non-functional genes present in the patients' cells with retroviral vectors constitutively expressing the therapeutical gene after random integration into the host genome. Consequently, the retroviral integration sites (RIS) create unique genetic signatures which can be amplified and sequenced to follow the fate of individual retrovirally ‘molecular marked’ cells and their clonal progeny in the respective target tissue [13]. Development of technologies for identification and sequencing of retroviral integration sites together with the publication of the human genome sequence have led to precise insights in the global integration pattern of different retrovirus subfamilies [14–20]. These integration site profiling studies uncovered unexpected virus specific integration preferences most likely resulting from different interactions of the viral integration machinery and host factors [21]. Comprehensive studies of retrovirally transduced cells and their fate *in vivo* by large scale integration site analyses of preclinical and clinical samples have provided evidence that the propensity for insertional mutagenesis is, at least in part, influenced by the non-random integration site selection of retroviral vectors [22].

In this chapter we will focus on recent insights in the target site selection of retroviral vectors and the molecular mechanisms underlying retroviral vector induced mutagenesis. An overview of subsequent biological effects of insertional mutagenesis is given based on preclinical and clinical data. We will introduce recent advances in next generation sequencing technologies and their impact on future high-throughput integration site analyses, both for mutation and vector biosafety research, and highlight their potential for a comprehensive clinical monitoring of current and future stem cell gene therapy trials using retroviral based vectors.

## 2. Trafficking to the Nucleus and the Retroviral Integration Reaction

Pioneering studies identifying the reverse transcriptase as a key component of retroviruses responsible for the conversion of the viral RNA genome into viral DNA [23,24] supported the provirus/protovirus [25,26] hypotheses by Howard Temin and led to the discovery of integrated vector genomes in many different organisms [27–32]. After penetrating the cell membrane, the viral nucleoprotein core particle containing two copies of viral genomic RNA is delivered into the cytoplasm where uncoating of the viral capsid takes place and reverse transcription is initiated [9]. Except for spumaviruses, which seem to have a unique mechanism to reversely transcribe their RNA genome late in the replication cycle [33,34], all other retroviruses initiate reverse transcription directly after endocytosis at the cell membrane [35]. The newly synthesized viral DNA remains in a large nucleoprotein complex called the pre-integration complex (PIC) and is associated with viral and cellular proteins [36–38]. The PIC interacts with the microtubule (MT) network that regulates intracellular trafficking to the nucleus [39–41]. Once at the nuclear membrane, gammaretrovirus based vectors such as MoMLV require the breakdown of the nuclear envelope during cell division to enter the nucleus [42]. In contrast, the PIC components of lentivirus based vectors [43] interact with the nuclear pore complexes allowing entry across the intact nuclear envelope [9]. Similar to yeast retrotransposons whose integration complexes interact with host proteins, interaction of the retroviral PIC with cellular proteins and their karyophilic properties support the tethering of the viral DNA genome to chromatin where the integration reaction is initiated [9,44].

The basic molecular mechanism of retroviral integration has been demonstrated in biochemical studies *in vitro* [45] and has been further elucidated by recent structural work [46,47]. The first steps in the integration reaction are catalyzed by the viral integrase and are initiated by the 3′-prime processing reaction which results in the removal of two nucleotides from each 3′-prime end of the viral DNA [45,48,49]. The exposed 3′-prime hydroxyl groups at each end of the viral DNA are joined to the target DNA and subsequent strand transfer reaction of a pair of processed viral DNA ends leads to a concerted insertion of the viral DNA into the host genome [45,48–50]. The sites of strand transfer on the two target strands are separated by 4–5 base pairs. Repair of this integration intermediate by cellular components [51] results in a direct duplication of 4–5 base pairs flanking the integrated viral DNA [2]. Most DNA sequences can act as integration acceptor sites, however, recent large scale studies on the integration site consensus of retroviruses *in vivo* [15,52,53] and *in vitro* [46,47] have shown that the base composition at retroviral target sites is biased for preferences or avoidances of particular bases supporting physical effects of the primary sequence on chromatin and the integration reaction, respectively.

### 2.1. Distribution of Retroviral Integration Sites in the Cellular Genome in vitro and in vivo

Since the discovery that integration is an essential step in the replication cycle of retroviruses, attempts were made to isolate proviruses and map their genomic location [2]. From *in vitro* studies using the purified integrase and recombinant chromatin it was suggested that nucleosome positioning influences the integration reaction [54–57]. Early studies on integration targeting of MLV in cultured cells proposed that integration was favored near DNaseI hypersensitive sites or transcribed regions [58–61]. However, due to the low number of individual insertion sites analyzed and the formerly unknown human genome sequence, target site selection of retroviruses across the whole genome was considered to be random.

The target site selection of retroviruses and retroviral vectors has gained novel scientific interest, since severe adverse events during the gene therapy trial for X-SCID [62] were linked to the integration of the therapeutic MLV based vector in vicinity of the LMO2 proto-oncogene [63,64]. Activating insertional mutagenesis of LMO2 in combination with acquired somatic mutations was responsible for the development of leukemia in a minority of the treated patients [65,66] and raised serious concerns whether the risk for insertional side effects is dependent on the target site selection and similar across all retroviral based vector systems [22].

**Figure 1:**
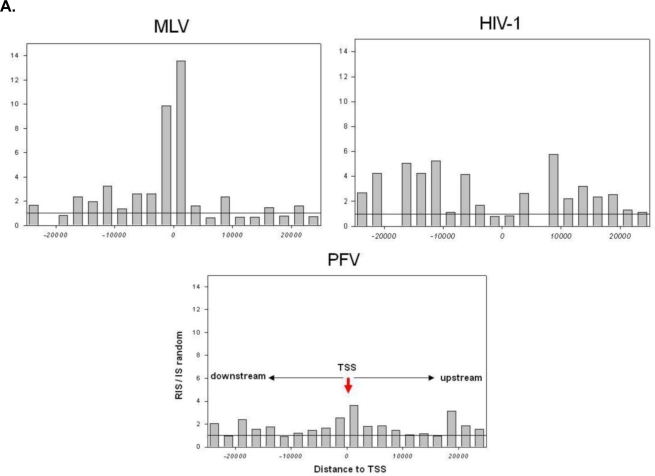

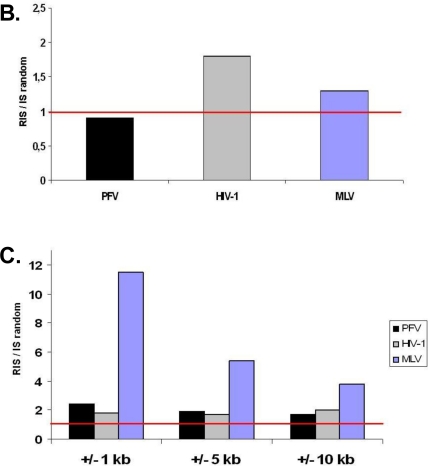
Integration site selection of retroviral based vectors in the cellular genome. (**A**) Analysis of individual vectors to target sites upstream and downstream of the transcription start site (TSS) at a 2.5 kb window size relative to the random control, which was set arbitrarily to 1 show that murine leukemia virus (MLV) based vectors have the strongest preference to integrate in close proximity to TSS. Prototype foamy virus (PFV) based vectors integrate at a rate approximately three- to four-fold higher than the expected random value, while HIV-based vectors avoid these regions; (**B**) The preference to integrate into transcribed regions of genes is strongest for HIV based vectors followed by MLV vectors, which show weaker preferences, while PFV vectors do not show any preferences for target site selection in genes. Their integration preference towards genes is similar to what would be expected if target site selection would be random; (**C**) Frequency of retroviral vectors to integrate in the vicinity of CpG islands matched to the random control, which was set arbitrarily to 1 is dramatically increased with MLV based vectors. HIV and PFV vectors do not show any strong preferences for integration near to CpG islands (modified from Nowrouzi *et al.* [11]).

With the decoding of the human genome and the development of PCR methodologies for allowing the amplification and sequencing of the genomic junctions between viral and host DNA the exact genomic location of a potentially unrestricted number of viral integrants generated by the acute infection of cultured cells with retroviruses or retroviral vectors became feasible. Analyzing their position in the human genome has uncovered virus specific integration patterns (Table 1) distinguishing most retroviral subfamilies from each other (Figure 1). The first large scale comparative analyses of integration target site selection in the human genome was performed with MLV and HIV-1 based vectors in a human cell line using LM-PCR and subsequent sequencing of the vector cellular junctions [19]. Compelling data demonstrated that RIS from MLV and HIV-1 based vectors showed distinct differences in respect to annotated features of the human genome. MLV vectors revealed a significant clustering around the transcription start site (TSS) of RefSeq genes and in the proximity to CpG-islands whereas HIV-1 based vectors disfavored TSS and CpG islands, but showed preferences for integration inside transcribed regions of RefSeq genes [19]. The preference for the integration of HIV-1 inside RefSeq genes was previously shown in a human lymphoid cell line [20] and also detectable with other vector systems from the lentiviral family pointing to a conserved mechanism of target site selection of lentiviruses [16,67,68]. These insights indicate that a mechanism involving the viral integrase and cellular genes is responsible for tethering the integration complex into particular regions of the genome. However, the investigated integration pattern of avian sarcoma and leukemia virus (ASLV) and prototype foamy virus (PFV) based vectors in human cells showed a rather random like distribution in relation to genes TSS and CpG-islands indicating that integration site selection of these retroviral families is not influenced by particular chromosomal structures and occurs rather by chance [14,15,18]. Based on changed integration patterns of HIV-1 hybrid vectors carrying the integrase gene of MLV it has been suggested that the principal viral determinant of retroviral integration specificity is the viral integrase itself [69]. The interaction of the HIV-1 integration complex with the cellular protein LEDGF/p75 plays a critical role in HIV-1 replication [70,71], protecting the viral integrase from proteosomal degradation [72] and increasing the affinity of the integrase to chromatin [73,74]. The siRNA mediated down regulation of LEDGF/p75 has been shown to influence the normal target site selection of HIV-1 based vectors decreasing its propensity to integrate into transcription units [75–78]. Very recently a comprehensive integration site analyses of HIV-1 based vectors in rodent eye and brain tissue revealed a decreasing preference for integration into transcription units correlating with a cell specific lowered expression level of LEDGF/p75 [79]. More intense investigations of different retroviruses and the functional analyses of cellular proteins interacting with their integration machinery will shed more light on the mechanism that retroviruses have evolved to maintain host cell genome integrity in accordance with virus specific replication and pathogenesis. The understanding of biochemical principles controlling the tethering of the retroviral integration complex to chromatin together with recent findings showing that such knowledge can be applied to redirect the integration pattern of HIV-1 based vectors are promising for the future development of therapeutic vectors which are designed to integrate into potentially safer sites of the genome [80–83].

The correlation of target site selection of HIV-1 based vectors to gene expression profiling data of virus infected cells has revealed that transcriptional activity of chromatin influences the genome accessibility of the retroviral integration complex [18,86]. Genes showing transcriptional activity have a higher propensity to be targeted by the HIV-1 integration complex [18]. Most likely, the transcriptional activity of chromosomal regions also plays a role in the expression of viral genes for wild-type retroviruses as well as transgene expression from retroviral vectors. Influences on the transgene expression level based on chromosomal regions have been demonstrated for HIV-1 based vectors with higher transgene expression from integrated vectors to be located on more active open chromatin [87]. From the evolutionary standpoint and pathogenesis of retroviral families it is interesting to note that ASV compared to HIV-1 seems to favor integration into more condensed chromatin *in vitro* similar to properties found at heterochromatin [88]; however, it remains elusive what mechanism controls these differences and if such differences might explain the different pathology of these retroviruses.

Recently, a genome wide analysis of >4000 transcription factor binding sites (TFBS) in the vicinity of MLV integration sites identified TFBS as differential genomic determinants of retroviral target site selection in the human genome. Gamma-retroviral vectors integrate in genomic regions enriched in cell-type specific subsets of TFBS suggesting that interaction of transcription factors with the viral LTR enhancer may synergize with the integrase in tethering retroviral pre-integration complexes to transcriptionally active regulatory regions [89]. A comparative analysis of gamma-retroviral integration sites between infused gene corrected mature lymphocytes (peripheral blood lymphocytes, PBL) and single infusion of hematopoietic stem/progenitor cells (HSC) of patients from the adenosine deaminase-severe combined immunodeficiency (ADA-SCID) clinical trial have revealed that the insertional profile of MLV based vectors is cell-specific according to the genetic/chromatin state of the target cell [90,91] indicating that retroviruses have developed different strategies to interact with the host chromatin which seem to influence integration site selection substantially [89,90].

To what degree does the different integration pattern of retroviruses and their vector derivates influence the likelihood for insertional mutagenesis? It is generally recognized that known preferences of gammaretroviral vectors to target regulatory regions of genes increase the risk for genetic alterations of neighboring genes [22,92]. In contrast, it has been shown that independent of the integration pattern also vector design and dose influence the likelihood of vector induced cellular transformation [93,94]. Virus specific transcriptional enhancers within the LTR have been identified as one of the major determinants of genotoxicity independent of retrovirus subtypes [93,94]. The deletion of enhancer/promoter elements within the retroviral LTR, termed self inactivating (SIN) LTR, significantly decrease the propensity to cellular transformation demonstrated in an *in vitro* immortalization assay [95]. However, at the same time it was elegantly shown in a tumor prone mouse model [96] that there is a significant higher load needed with LV vectors containing gammaretroviral enhancer elements to trigger oncogenesis when compared to MLV-based vectors [93,97]. The authors suggest that the higher vector load required for oncogenesis by LV vectors is most likely explainable by a higher preference of MLV-vectors to integrate near cancer promoting genes pointing to a role of intrinsic viral integration patterns inducing genotoxicity. Initial studies using PFV-vectors with rather random like integration patterns with respect to genes and regulatory regions of genes in a canine preclinical model have not resulted in any detectable insertional induced side effects [98], arguing that random and uniform integration is safer in terms of genetic alterations of the host genome.

Taken together, many large scale integration site distribution studies have been conducted and contributed to a better understanding of factors which may influence integration site preferences of retroviral vectors. Potential genetically safer vector systems, based on their integration target site selection, have been evaluated by several groups showing that deletion of viral genotoxic elements and the use of physiological (cellular) promoters significantly reduce the risk for insertional mutagenesis in future gene therapy trials.

### 2.2. Next Generation Sequencing and Unbiased Retrieval of Vector Integration Sites

**Figure 2:**
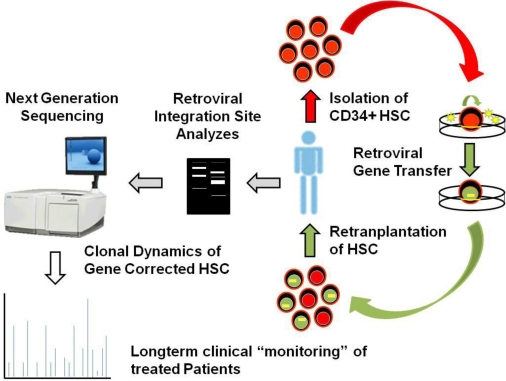
Analyzing clonal dynamics and insertional induced side effects by retroviral integration site analyzes and next generation sequencing.

For characterization of proviral cellular junctions two fundamental PCR methods were developed: inverse PCR [99] and ligation-mediated PCR [100–102]. Although further development in these methodologies led to improvements in sensitivity, the invention of the linear amplification-mediated PCR (LAM-PCR) [103,104] enabled the characterization of integration sites at the single-cell level for the first time, allowing monitoring of retrovirally gene-modified hematopoiesis directly in limited amounts of peripheral blood leukocytes and bone marrow cells [105]. Combined with next generation sequencing platforms and strategies for saturated genomic access [106–108] LAM-PCR has proven to be highly efficient for retrieval of whole insertional inventories in clinical and preclinical samples [109] (Figure 2) and analyzing the clonality of the hematopoietic repopulation after transplantation in humans [66,110–114].

The principle of LAM-PCR has been described previously in detail [103]. The first step is the pre-amplification of the vector-genome junctions by a linear amplification step with biotinylated primers hybridizing at one end of the integrated vector. The following steps are carried out on a semisolid streptavidin phase in order to capture DNA strands with an incorporated biotinylated vector primer. After double strand synthesis, a restriction digest, and the ligation of a linker cassette on the genomic end of the fragment, two exponential PCRs with nested arranged vector- and linker cassette primers are carried out in order to amplify the fragments consisting of linker cassette-, genomic-, and vector-sequence. The generated fragments are sequenced and the integration loci are determined. Application of novel next-generation sequencing technologies for integration site analysis by several groups have led to a marked increase in the retrieval of integration site information. We have recently applied 454 pyroseqeuncing and annotation of restriction motives in the human genome to define the genomic accessibility to viral integration sites and developed a new modified protocol for unbiased retrieval of insertion sites based on the non-restrictive LAM-PCR (nrLAM-PCR) [106,107]. The nrLAM-PCR allows the genome-wide identification of retroviral integration sites in a single reaction, circumventing the detection bias accompanied by methods dependent on restriction enzymes [106,107]. Multiplex barcoding [115] of samples together with downstream bioinformatical evaluation of up to 1 million 400 bp long reads in one sequencing run enables analyzing over hundred thousand RIS in multiple samples at the same time [107]. This is efficient to analyze whole insertional inventories of patients, clonal dynamics and integration patterns of any kind of integrating genetic elements in parallel. Most importantly, LAM-PCR or nrLAM-PCR combined with next generation sequencing platforms (*i.e.*, 454, Solexa, ABI) allow a semi quantitative retrieval of insertion sites representing unique molecular identities of particular marked clones in a given sample, highly valuable to monitor clonal dynamics in hematopoeisis and many other biological systems [110,114,116–118]. Several commercial sequencing platforms are available, each having its advantages and disadvantages. The platforms from Solexa (Genome Analyzer) and Applied Biosystems (SOLID) generate very high numbers of sequence reads, but are limited in individual sequence length. The pyrosequencing platform from 454 Life Sciences (Titanium system; Roche Diagnostics) allows the generation of up to 5 × 10^5^ sequence reads of ∼400 base pairs (bp) in length in a single sequencing run sufficient to chromosomal map amplicons with vector cellular junctions without using complicated algorithms to process short sequence reads.

## 3. Side Effects in Clinical and Preclinical Gene Therapy Studies

**Figure 3:**
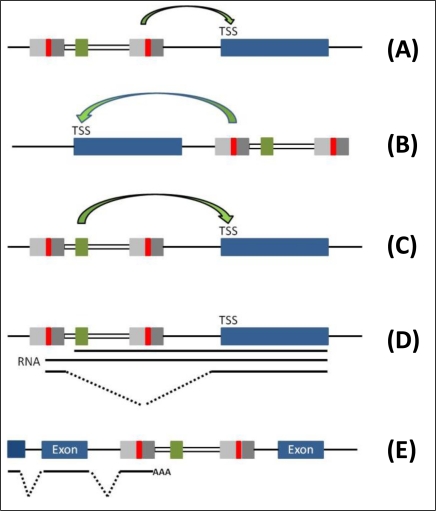
Retroviral vectors may induce mutations in multiple ways by integration of the retrovirus in the host genome. (**A**, **B**) Mutagenic proviral insertions in most reported cases induce an activation of neighboring genes by enhancer elements present within the wildtype LTR. Such “enhancer insertions” can induce gene activation from distances up to 100 kb. (**C**, **D**) In case of SIN-type retroviral vectors strong internal promoters driving transgene expression may induce deregulation of genes in close proximity similar to so-called “promotor insertions” which result in viral-host gene-fusion transcripts. (**E**) Genotoxic side effects resulting from retroviral integration sites leading to inactivation of cellular genes may be induced by viral insertion within a host gene leading to truncated non functional transcripts.

First molecular insights in the oncogenic potential of integrated wild type retroviruses were obtained from bursal lymphomas in chicken. The majority of the identified tumor cells contained a provirus integrated in the vicinity of the proto-oncogene c-myc that was overexpressed by the viral promoters/enhancers [119–122]. These genetic alterations caused by retroviruses, termed proviral insertional mutagenesis, have been identified in many types of retrovirus induced tumors [123,124]. Thereby, a variety of genes could be identified that modulate growth and differentiation and significantly contributed to tumor formation. Despite these insights obtained from wild-type retroviruses and replication-competent retroviral vectors, the risk of insertional mutagenesis (Figure 3) and cellular transformation with replication-incompetent vectors specifically developed for clinical purposes was considered to be rather low [125].

However, after first cases of severe adverse events in a minority of patients in the X-SCID gene therapy trial were reported (see next chapter), the first malignant transformation that developed as a result of gene transfer using replication-deficient retroviral vectors designed for clinical use in a murine model was observed in 2002 [126]. In a murine model system the serial transplantation of bone marrow cells marked with gammaretroviral vectors led to tumor development in secondary and tertiary recipients [126]. All secondary transplanted animals showed alterations in their hematopoiesis after 22 weeks, and six out of ten animals developed acute myeloid leukemia. Molecular analyses of the malignant clone in respect to the RIS using LM-PCR revealed that in all developed tumors derived from secondary and tertiary recipients an insertion in the *Evi1* gene locus triggering malignant transformation was detected [126]. Although it was shown that the promoter and enhancer elements in the proviral LTR caused an overexpression of Evi1, a synergistic effect of the low-affinity nerve growth factor receptor (LNGFR) remains unclear [126]. In terms of clinical safety it is important to note that vector dose has an impact on leukemogenesis as shown in a murine study where Leukemia not only occurred due to the growth advantage of single clones but also correlated with high vector doses using MLV based vectors [127]. Soon after, gene marking studies in a non-human primate model provided evidence for clonal dominance due to an insertional effect of MLV based vectors in vicinity of the Mds1/Evi or Prdm16 gene. This insertional event led to a growth advantage compared to other marked cell clones without causing malignant transformation [128]. Since the majority of these cell clones contribute to long-term hematopoiesis, a higher engraftment or survival probability as a result of insertional mutagenesis was discussed [22,128]. Vector induced side effects with possible roles in hematopoietic activity have also been reported in a murine gene marking study in which proviral integration within or nearby particular cellular genes promoted the growth of single transduced cells and contributed to their clonal expansion *in vivo* [129]. The first retroviral vector induced acute myeloid leukemia in a non-human primate model was described after retroviral gene transfer in hematopoietic precursor cells. The treated animal died five years after gene therapy due to a myeloid sarcoma caused by two insertions in the *Bcl2-A1* and *Cdw91* genes. In this study the clone harboring these two insertions was both detectable in the blood where it became dominant one year after transplantation as well as in the tumor, strengthening a cooperative functional role of these insertions in tumor development [130].

Human gene therapy using retroviral vectors is a specialized form of therapy that is mainly applied to patients for whom no therapeutic alternatives are available. Therefore, the benefit of gene therapy always has to be opposed to its potential risks. The field has suffered from a lot of initial hype without taking into account that any new kind of specialized therapy may also be accompanied by side effects. The success of human gene therapy for hematopoietic diseases has been enabled by the greater efficiency in performing *ex vivo* transduction of human CD34^+^ cells using retroviral vectors and reinfusion of a high number (up to 10^8^−10^9^) of gene-corrected cells. In retrospect, it is now evident that improvement in effectiveness for curing otherwise challenging lethal diseases is accompanied by a higher risk of biologically relevant side effects of this type of gene therapy. Up until now most gene therapy trials in the hematopoietic system involve the use of first generation MLV derived retroviral vectors. Classical MLV based vectors carrying the full LTR are now based on their integration site selection and vector design considered to have a higher potential for genotoxic events as compared to lentiviral based vectors (see below).

Monitoring the *in vivo* fate of gene corrected CD34^+^ cells retransplanted into patients by high-throughput integration site analysis in five independent clinical gene therapy studies have shown that distribution of gammeretroviral vectors *in vivo* is skewed. Apart from subtle effects, clonal dominance and leukemogenesis influencing the growth and differentiation of clones when inserted near to particular genes and loci have been reported in minority of treated patients [64–66,111–114,131]. We have recently described that in addition to overt carcinogenesis, even as a single vector copy, many insertion locations more subtly influence the biological fate of a cell clone *in vivo* [111,112,123]. With LAM-PCR technology specifically developed for this purpose, it has been identified that up to 40% of circulating cells carry insertions in a rather small set of frequently affected common insertion sites (CIS) [111,112]. Such CIS are almost always in the direct vicinity of known and novel genes likely to be involved in cellular growth, survival and self-renewal processes of immature progenitor and stem cells.

In the gene therapy trial for X-SCID, two to six years after successful correction of the otherwise lethal disease the development of T-cell leukemia in five of 19 patients has been observed [65,66]. In four of these patients the leukemic clone harbored a vector integration in the *LMO2* proto-oncogene inducing its overexpression. Together with acquired mutations these events have led to expansion of these clones and the development of leukemia [65,66]. Although under dispute, the combinatorial effect of the *IL2RG* transgene in the development of the lymphoproliferative disease in the X-SCID trial has been discussed [132,133] and recent work from Copeland and colleagues provides supportive evidence that *IL2RG* and *LMO2* do cooperate in leukemia induction [134]. At the same time skewing of RIS was observed in the healthy patients causing subtle insertional effects without any signs of malignant transformation [111,112].

**Figure 4:**
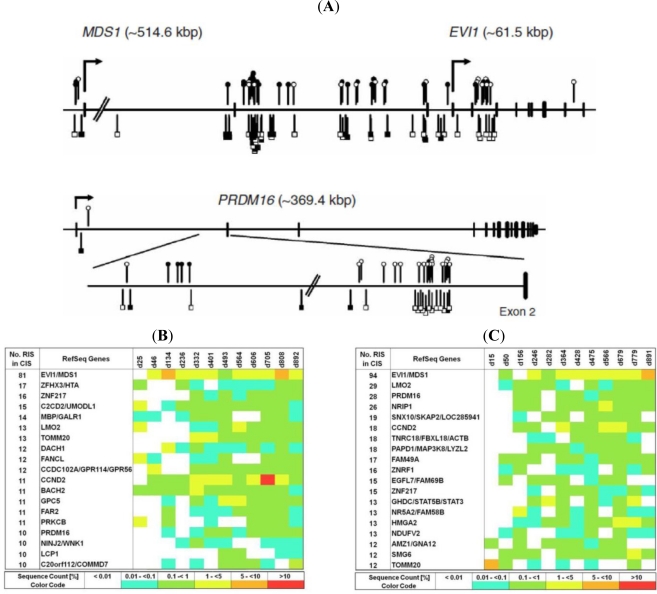
Retroviral integration into common insertion sites in clinical gene therapy trials. (**A**) Clustering of retroviral integration sites (RIS) within clones sharing integrations in MDS1-EVI1, PRDM16 and SETBP1 in the X-CGD clinical trial identified by linear amplification-mediated PCR (LAM-PCR) (modified from Ott *et al.* [113]). (**B**) Clinical monitoring of retroviral gene corrected hematopoietic stem/progenitor cells (HSC) by LAM-PCR and 454 sequencing in two patients from the WAS gene therapy trial over time reveal multiple clones sharing insertion sites into common integration sites located near genes previously known to induce malignant clonal expansion. For measuring clonal contribution to hematopoiesis over time at every time point analyzed, sequence counts for all RIS contributing to an individual common insertion sites (CIS) derived from PBL and BM were clustered and related to total sequence count at the respective time point (modified from Boztug *et al.* [114]).

Evidence for vector induced effects influencing hematopoietic activity have been gained in the gene therapy for the correction of X-CGD [113,117]. Here, in two adult patients, retroviral gene therapy resulted in the restoration of oxidative antimicrobial activity in phagocytes after gene transfer. Integration site analysis revealed a clonal dominance triggered by insertion sites in the genes *MDS1-EVI1*, *PRDM16* or *SETBP1* (Figure 4A), resulting in an expansion of gene corrected myelopoiesis [113,117]. In the follow up of the clinical trial a substantial gene transfer in neutrophil cells had produced a high number of functional phagocytes, however, after the initial resolution of bacterial and fungal infections, both subjects showed silencing of transgene expression due to methylation of the viral promoter, and myelodysplasia with monosomy 7 as a result of insertional activation of ecotropic viral integration site 1 (*EVI1*). It has been recently suggested that the overexpression of Evi1 disrupts normal centrosome duplication leading to genomic instability, monosomy 7 and clonal progression toward myelodysplasia [117].

From these severe adverse events it is tempting to reason that the use of gammaretroviral vectors is *per se* oncogenic. However, given that severe events have occurred in a minority of treated patients in the X-SCID trial, the clinical follow up of the ADA-SCID [131] trial using the same vector backbone as in the X-SCID trial has up to now not been accompanied by any adverse events up to 8 years post therapy. This may indicate that the risk for vector induced side effects may also be dependent on the disease and the clinical protocol. We note that clear evidence for this discussion has not been reported so far.

In the most recent gene therapy trial for Wiskott-Aldrich Syndrome (WAS) an MLV-based vector backbone expressing the WAS protein was used for transduction of autologous CD34^+^ HSC which were transfused into two patients. WAS is a primary immunodeficiency disorder associated with thrombocytopenia, eczema and autoimmunity and is alternatively treatable with haploidentical BM transplantation [114]. Here, monitoring the clonal contribution of gene corrected cells to hematopoietic regeneration three years post transfusion by a comprehensive integration site analysis using next generation sequencing technologies has identified over 10,000 unique clones contributing to short- and long-term hematopoieses. This genome-wide insertion site analysis demonstrated that vector integration targeted multiple genes controlling growth development and immunological responses in a persistently polyclonal hematopoiesis. However, many of the previously observed CIS, which occasionally triggered adverse events, were detectable in both subjects to a similar degree suggesting vector induced skewing similar to what was observed in previous gene therapy trials using the MLV backbones (Figure 4B, C). In fact, it was further shown that in sorted cell populations of the lymphoid or myeloid fraction lineage specific CIS could be identified, which in previous gene therapy trials had triggered either lymphoid or myeloid proliferation suggesting that insertional activation of these genes programs cell fate in the hematopoietic system [114]. In total, 9/10 patients have been treated successfully in this trial. Very recently it has been reported that one patient has developed a T-cell leukemia similar to the patients in the X-SCID trial. Whether vector induced effects have played a role in this side effect is under current investigation but it is most likely that vector induced side effects similar to the side effects observed in the minority of X-SCID patients may play a role here.

First proof that switching the integration pattern of the therapeutical retroviral vector improves genotoxic safety has been provided by the first LV-based gene therapy trial for the treatment of the cerebral form of X-chromosomal linked adrenoleukodystrophy (X-ALD), a demyelinating disease of the central nervous system caused by mutations in the *ABCD1* gene [110]. Progress of X-ALD is treatable by haploidentical BM transplantation, but with patients where no matching donor is found gene therapy is a highly promising therapeutic option [135]. In 2006, two patients with no sibling BM donors were treated with HIV-1 based vectors, which show distinct differences in target site selection and efficiency in transducing quiescent cells compared to MLV-based vectors. Mobilized hematopoietic precursor cells were harvested from two seven year-old patients, transduced *ex vivo* with lentiviral SIN-vectors encoding the *ABCD1* transgene and reinfused into the patients. This treatment proved efficient to arrest the progression of the disease in both patients through the constitutive expression of the functional *ABCD1* therapeutic transgene. More importantly, no signs of vector induced side effects have been reported so far in two treated patients continuously being monitored. Up to now, the clonal dynamics of transduced and reinfused CD34^+^ HSC show transduction of multipotent HSC and no signs of enrichment near to CIS genes previously being identified as integration hot spots of MLV-based vectors *in vivo* [110]. The improved clinical safety of SIN-type LV-based vectors compared to classical MLV-based vectors has previously been proposed based on evidence gained in sensitive mouse models designed to measure genotoxic safety [93,136]. Deduced with murine genotoxicity assays, although at low rate, vector induced genetic alterations have also been reported with target site selection of LV-based vectors [93,94,137]. Such insertional alterations in the regulation of the cellular genome by LV-vectors have recently been reported in the gene therapy trial for human β-thalassaemia [118]. Clinical benefit of lentiviral β-globin gene transfer has been achieved in an adult patient 33 months post treatment by clonal dominance initiated by vector induced activation of the *HMGA2* gene. This particular clonal dominance has been proposed to accompany clinical efficacy for β-thalassaemia, a challenging hematopoietic disease, and the most common form of severe thalassaemia in southeast Asian countries and their diasporas [118].

### New Strategies for Vector Biosafety in Gene Therapy

The risk of side effects in future gene therapy trials will be dependent on the choice of the most suitable gene transfer vector dependent on the individual disease and the propensity for genomic alterations driven by vector design and integration pattern. The usage of SIN-type vectors with weak or tissue-specific promoters or non-integrating vector systems—if appropriate—are considered to be safer than classical MLV-vectors. The use of AAV- or Adenovirus-vectors predominantly persisting episomaly in post-mitotic tissue (*i.e.*, retina, brain, muscle or liver) are promising for particular diseases [138]. However, insertional mutagenesis caused by rare but detectable integration events with AAV-vectors in the liver has also been reported [139,140] underlining the need for highly sensitive strategies to detect such rare insertions in a clinical setting. In postmitotic tissues, dilution of unintegrated episomal vector forms does not occur thus allowing the use of integrase deficient lentiviral vector systems [141]. Mutations in the core domain of the viral integrase prevent integration, thereby reducing the risk of insertional mutagenesis. Recently, the long-term functional correction of retinal degeneration in a well-established rodent model for ocular gene therapy was reported under the use of an integrase-deficient HIV-1 vector without apparent side effects [142].

Alternative modification of the viral integrase for targeted integration into desired potentially safe chromosomal regions [143], together with significant improvements in the efficiency of homologous recombination or gene disruption using novel Zinc-Finger-Nucleases (ZFN) will in future define alternative gene delivery approaches [144]. Other non-viral integrating vector systems based on sleeping beauty or piggybac transposons have been identified to have a potentially safer integration pattern *in vitro* [145]. However, given that any integrating vector system by chance, if combined with genotoxic vector elements, may induce genetic alterations of the cellular genome also account for transposon based vectors which are also used to identify novel cancer genes in murine models [146].

With significant improvements in the controlled genetic modification of particular loci in targeted integration approaches and a variety of vectors with alternative integration patterns available it becomes evident that the advance of highly sensitive technologies for a comprehensive genomic screening is required to monitor safety on a genetic level. Whole genome next generation sequencing technologies to evaluate genomic stability and strategies for an unbiased retrieval of integrated sites [106] and induced DNA double-strand breaks will be essential for dissecting the genetic specificity and safety of novel targeted gene editing protocols. In this context, recent pioneering advances in the field of cellular reprogramming and the availability of sources of induced pluripotent cells (iPS) [147–149] or lineage specific progenitor cells [150–152] generated by integrating vector systems expressing particular pluripotency of lineage regulating transcription factors have made prediagnostic genomic safety screening of hundreds of clones feasible for potential future cell based therapies [153]. New vector systems and the potential use of new induced clinical applicable cell sources will greatly benefit from vector biosafety models established in the gene therapy field [154,155].

During the last years several *in vitro* and *in vivo* systems have been developed assessing the genotoxic potential of different integrating vectors. Transplantation of gammaretroviral transduced hematopoietic cells in mice with knocked-out *Cdkn2a* tumor suppressor gene leads to accelerated tumor growth in case a cellular proto-oncogene is virally activated [96,156]. Such conducted insertional mutagenesis screens have resulted in the establishment of the most extensive database of murine genes with oncogenic potential, the so-called mouse “*Retrovirally Tagged Cancer Gene Database*” (RTCGD) [157]. Recently also sleeping beauty transposon systems have been applied in a similar manner [158,159]. Du and colleagues showed that identification of protooncogenes is feasible by retroviral gene transfer in cell culture. Transduction of murine bone marrow cells with replication deficient retroviruses expressing marker genes resulted in immortalized cell lines, many of which contained integrations in the *Mds1/Evi1* and *Prdm16* genes [160,161]. Vector integration into the *Evi1* locus and overexpression of *Evi1* appeared to be sufficient for a cell to be immortalized [161,162]. Modlich and colleagues adapted and improved this method so that the results could be analyzed in a quantitative manner [95].

The tumor prone mouse model in which the tumor suppressor gene *Arf* as well as the *Il2rg* gene had been knocked-out originally developed by Lund *et al.* was suitable to perform comparative tests on the genotoxic potential of different gene transfer vectors [96,97]. Mice that had received hematopoietic precursor cell transplants transduced with gammaretroviral vectors developed tumors much more rapidly than mice that had received cells harboring lentiviral SIN-vectors with an internal human phosphoglycerate kinase (hPGK) promoter [97]. Later, it was demonstrated that gammaretroviral vectors with SIN-LTRs show a significant reduction in genotoxicity, but when combined with strong (viral) internal promoters still have the ability to induce leukemia in transplanted mice [93]. Given that in follow-up of the first LV-vector gene therapy trial for X-ALD no signs of clonal dominance or side effects have been observed up to the present it seems reasonable that such mouse models realistically modulate clinical safety of integrating vector systems. Insertion of insulator elements in the LTRs aims to reduce the influence of the integrated vector on the surrounding host genome and *vice versa* [163,164]. However, evidence for increased genotoxic safety with the use of insulators [165] needs to be further provided in common *in vitro* and *in vivo* models for vector safety assessments, especially after insertional mutagenesis mediated clonal dominance has been reported in the lentiviral β-thalassaemia gene therapy trial in which the cHS4 chromatin insulator was implanted in the LTR of the therapeutic vector [118].

Improvement of genotoxic safety using alternative and modified vector systems together with progress in targeted integration approaches and new cell sorting abilities provide a tremendous potential for future gene and cell therapy with increased safety. This will further improve the treatment of patients with otherwise not curable and often lethal inherited diseases.

## 4. Conclusion and Future Perspective

Large scale genome wide investigations of retroviral target site selection have shown that different retroviral subfamilies have developed different mechanisms to integrate their DNA into the host genome. Such differences result in three main integration patterns with MLV based vectors showing strong preferences for regulatory regions, LV-based vectors integrating preferentially inside RefSeq genes and PFV as well as ASLV having close to random insertional preferences. Towards safe clinical application of retroviral vectors uncovered target site preferences have proven to play substantial roles in the likelihood for insertional mutagenesis and cellular transformation. Knowledge gained from comprehensive integration site analysis of gene therapy patients and preclinical animal models to assess genotoxic safety of retroviral vectors are currently being actively transferred into the development of new and safer clinical protocols for the treatment of hematological and neurodegenerative diseases. The vision of using vectors with targeted synthetic integrases and improving the frequency of homologous recombination with ZFN or Meganucleases in order to correct defective genes or insert therapeutic genes into potentially “safe harbors” is rapidly evolving to become clinically feasible. In the era of whole genome sequencing projects aiming to understand progress of cancer and other diseases on a genetic level, the treatment of many new diseases in which the pathogenesis is dependent on a gene defect may become treatable with novel safe retroviral vectors imposing minimal genomic side effects for the patients.

## Figures and Tables

**Table 1:** Integration pattern of different retroviral vectors.

**Virus**	**Genes**	**TSS**	**CpG-Rich Islands**	**Reference**
HIV	+	−	−	[19,20]
SIV	+	−	−	[67]
MLV	±	+	+	[18,19,67]
ALV	±	−	−	[17]
ASLV	±	−	−	[18]
PFV	−	±	±	[14,15]
EIAV	+	−	±	[16,68]
HTLV-1	±	−	−	[84,85]

(+) strong preference;

(−) no preference;

(±) weak preferences.
